# Household food insecurity and children’s physical activity and sedentary behaviour in the United States: the Healthy Communities Study

**DOI:** 10.1017/S1368980021002536

**Published:** 2022-02

**Authors:** Sophia M Navarro, Marisa M Tsai, Lorrene D Ritchie, Edward A Frongillo, Barbara A Laraia, Russell R Pate, Lauren E Au

**Affiliations:** 1 Nutrition Policy Institute, Division of Agriculture and Natural Resources, University of California, Oakland, CA, USA; 2 Arnold School of Public Health, University of South Carolina, Columbia, SC, USA; 3 School of Public Health, University of California, Berkeley, Berkeley, CA, USA; 4 Department of Nutrition, University of California, Davis, 3215 Meyer Hall, One Shields Avenue, Davis, CA 95616, USA

**Keywords:** Food insecurity, Physical activity, Children, Obesity

## Abstract

**Objective:**

To examine associations between household food insecurity and children’s physical activity and sedentary behaviours.

**Design:**

Secondary analysis was conducted on the Healthy Communities Study, an observational study from 2013 to 2015. Household food insecurity was assessed by two items from the US Department of Agriculture’s 18-item US Household Food Security Survey Module. Physical activity was measured using the 7-d Physical Activity Behaviour Recall instrument. Data were analysed using multilevel statistical modelling.

**Setting:**

A total of 130 communities in the USA.

**Participants:**

In sum, 5138 US children aged 4–15 years.

**Results:**

No associations were found for the relationship between household food insecurity and child physical activity. A significant interaction between household food insecurity and child sex for sedentary behaviours was observed (*P* = 0·03).

**Conclusions:**

Additional research capturing a more detailed assessment of children’s experiences of food insecurity in relation to physical activity is warranted. Future studies may consider adopting qualitative study designs or utilising food insecurity measures that specifically target child-level food insecurity. Subsequent research may also seek to further explore sub-group analyses by sex.

In 2019, 13·7 million households in the USA experienced food insecurity^(1)^. Among households with children, 2·4 million had children who were food insecure^(1)^. High prevalence of food insecurity was observed in households with children (13·6 %) and those headed by single mothers or single fathers (28·7 % and 15·4 %, respectively). Additionally, non-Hispanic Black households, Hispanic households and low-income households below 185 % of the poverty line were also observed to have high prevalence of food insecurity (19·1 %, 15·6 % and 27·6 %, respectively)^(1)^. The prevalence of household food insecurity was highest for those living in the Southern USA (11·2 %) and in the principal cities of metropolitan areas (12·4 %)^(1)^.

Food insecurity is associated with poorer physical health, cognitive development, academic achievement and behaviour in children and has been linked to higher odds of childhood obesity^(2–4)^. Risk of childhood obesity is also associated with physical activity and sedentary behaviours^(5,6)^. Few studies have investigated the connections between food insecurity and physical activity in children, however, and those that have explored this link demonstrate mixed associations, with some finding negative associations with moderate-to-vigorous physical activity, null or negative associations with sedentary behaviour and null associations with total physical activity, defined as the sum of light, moderate and vigorous intensity physical activity^(7–9)^. It is hypothesised that food insecure children may be less likely to participate in physical activity because food insecurity can have physical (e.g. hunger, fatigue and illness) and emotional (e.g. stress, anxiety and depression) manifestations^(10,11)^. Thus, because both food insecurity and inadequate physical activity can result in serious health consequences and interventions aimed at decreasing food insecurity may positively impact physical activity, it is critical to examine this relationship in children. This research will help clarify the relationship between food insecurity and physical activity, contributing meaningful insights into the implications of food insecurity in children.

The primary aim of this study was to examine whether household food insecurity is associated with children’s physical activity and sedentary behaviour. Prior studies have indicated that factors influencing physical activity may operate inconsistently across certain subgroups, such as age, sex and race/ethnicity. For example, trends in physical activity behaviour among US youth reveal that children aged 12–15 years tend to be less physically active than children aged 6–11 years, high-school girls tend to be less physically active than high-school boys and there are reported differences in activity by race/ethnicity^(12)^. Thus, this study will also explore whether the relationship between food insecurity and physical activity differs within specific subpopulations, including age, sex and, race/ethnicity.

## Methods

### Study design and participants

The Healthy Communities Study is a cross-sectional observational study of 5138 US children designed to assess the impact of community programmes and policies addressing childhood obesity on child diet, physical activity and weight outcomes. Participants included those 4–15 years in grades K-8 and were chosen from a diverse sample of 130 communities, defined as high-school catchment areas, across the country between 2013 and 2015. Community selection was based on hybrid sampling that included a national probability-based sample of 102 communities as well as a sample of twenty-eight ‘certainty’ communities known for having programmes and policies that target childhood obesity prevention. Data collectors interviewed children and families in their homes, gathering comprehensive information on demographics, nutritional status, anthropometry, physical activity and the surrounding environment. The Battelle Memorial Institute Institutional Review Board approved the study protocol, and parents provided written informed consent for child participation. A more detailed explanation of participant recruitment, study design and data collection are included in papers by Sagatov *et al.*, Strass *et al.* and John *et al.* ^(13–15)^.

### Variables of interest

Demographic information was provided by the parent or guardian and included items on child age, child race/ethnicity, geographic region, urbanicity, country of origin, household income, parental education attainment, marital status and employment status. Household food insecurity was indicated by two items from the US Department of Agriculture’s 18-item US Household Food Security Survey Module that assess uncertainty that the household food budget or food supply is sufficient to meet basic needs^(16)^. The indicator from these two items has high sensitivity (97 %) and specificity (83 %) in predicting responses to the full module and are associated with poor or fair child health, hospitalisations and developmental risk^(17)^. Furthermore, any affirmation of household food insecurity is associated with children’s academic performance, weight gain and social skills^(18)^. Administered by an interviewer, respondents were asked to assess how often the following occurred within the past 12 months: (1) ‘We were worried whether our food would run out before we got money to buy more’ and (2) ‘The food we bought just didn’t last and we didn’t have money to get more.’ Participants could respond with *very often, often, sometimes, rarely* or *never*. Those who responded with *very often, often* or *sometimes* for at least one of the items were categorised as having household food insecurity. For all households, the adult was the respondent.

Physical activity was measured using self-report in the 7-d Physical Activity Behavior Recall (PABR-7) instrument. The tool was designed to detect forms of physical activity and sedentary behaviour that are typically targeted in community interventions to reduce childhood obesity. Through a computer-assisted interview, respondents specified whether the child participated in each activity over the past 7 d, the days the activity was performed and the average intensity of the activity (i.e. light, moderate, hard and very hard) using sex- and age-appropriate visual aids. For children aged 4–8 years, their parent/guardian responded, and the child was there to assist. For children aged 9–15 years, the child responded, and their parent/guardian was present to assist^(19)^. In this study, physical activity terms refer to the frequency and intensity of specific forms of activity and are therefore used in a manner that is specific to the methodology of the study.

Four measures were used to assess a child’s physical activity: total physical activity, vigorous physical activity, moderate-to-vigorous physical activity and sedentary behaviours. These variables were converted into index scores by multiplying the number of reported activities for each activity intensity level by the frequency of participation in those activities. Greater scores indicate more physical activity and sedentary behaviour. The total physical activity index score included fifteen activities: PE class, recess/free play, dance, activity breaks, school sports, non-school sports, pick-up sports, physical activity in after-school, physically active games, swim/water activities, outdoor/adventure activities, walk/bike to school, walk/bike to store/friend’s house/etc., walk/bike for fun/exercise and active video games. The vigorous physical activity index score included four activities: school sports, non-school sports, pick-up sports and outdoor/adventure activities. The moderate-to-vigorous physical activity index score included eleven activities: PE class, recess/free play, dance, school sports, non-school sports, pick-up sports, physical activity in after-school, physically active games, swim/water activities, outdoor/adventure activities and walk/bike for fun/exercise. The sedentary behaviours index score included four activities: used a computer for games or playing on the internet (not for schoolwork or social networks), used a computer or phone for social networking, watched TV and played non-active video games^(19)^.

### Data analysis

Secondary data analysis was conducted on the Healthy Communities Study data set. Participant demographics were analysed by household food insecurity status using t-tests for continuous variables and *χ* ^2^ tests of independence for categorical variables. Multilevel linear regression models were generated to relate food insecurity with physical activity, adjusting for child- and community-level covariates and clustering by school and community levels. Multiple imputation by chained equations was used to account for missing data due to participant nonresponse whereby twenty imputed data sets were created and then combined^(20)^. Approximately a quarter of cases (24·5 %) were missing at least one variable. The least absolute shrinkage and selection operator (LASSO) technique was used to select a subset of covariates from the large pool of demographic variables available, increasing accuracy and interpretability of models^(21)^. The covariates included in the models were maximum parental education, maximum parental employment status, child age, child age squared, child race/ethnicity, seasonality, US region, income classification, community percentage of African Americans, community percentage of high school graduates and community unemployment rate for those in the labour force 16 years and older.

Additional, exploratory multilevel linear regression models were generated to assess for interaction with child age, sex and race/ethnicity. Child sex was coded as female or male, based on the identification by the data collector. The race/ethnicity variable had four options: Non-Hispanic White, Non-Hispanic African American, Non-Hispanic other and Hispanic or Latino. Based on a review of the relevant literature assessing how physical activity varies by age, age was divided into 4–11 years and 12–15 years^(12)^. All analyses were conducted in SAS 9.4 (SAS Institute). Reported *P*-values are two-sided, and significance was set at *P* < 0·05.

## Results

In this study sample, children were on average 9 years old and half were female (Table 1). Nearly half of children were Hispanic or Latino (44·7 %). The majority (72·9 %) had at least one parent working full-time; less than half of parents (42·9 %) had a maximum education of high school or less. Geographically, 41·6 % of participants were from the Southern USA. At the community level, nearly 19·7 % of children lived in a community with a census tract minority classification of African American and 34·8 % with an income classification of low income^(22,23)^.

**Table 1:** Socio-demographic characteristics of the children in the Healthy Communities Study by household food insecurity status (*n* 5138)

Characteristic*	Total *n* 5138		Without household food insecurity *n* 2845 (55·4 %)		With household food insecurity *n* 2293 (44·6 %)		*P*-value†,‡
Child and household characteristics							
	*n*	%	*n*	%	*n*	%	
Child sex							<0·001
Female	2614	50·9	1390	48·9	1224	53·4	
Child race/ethnicity§							<0·001
Non-Hispanic white	1520	29·6	1177	41·4	343	15·0	
Non-Hispanic African American	927	18·0	434	15·3	493	21·5	
Non-Hispanic other	396	7·7	271	9·5	125	5·5	
Hispanic or Latino	2295	44·7	963	33·9	1332	58·1	
Maximum parental employment status||							<0·001
Full-time	3747	72·9	2340	82·3	1406	61·3	
Part-time	518	10·1	217	7·7	299	13·1	
Unemployed	273	5·3	87	3·0	185	8·1	
Other	600	11·7	199	7·0	401	17·5	
Maximum parental education¶							<0·001
Less than high school	1164	22·7	390	13·7	772	33·7	
High school diploma	1036	20·2	425	14·9	611	26·7	
Some college or Associate degree	1282	25·0	755	26·5	629	27·5	
Bachelor’s degree	787	15·3	594	20·9	193	8·4	
Graduate degree	869	16·9	783	27·5	86	4	
	Mean	sd	Mean	sd	Mean	sd	
Child age in years	9·3	2·7	9·2	2·7	9·5	2·6	<0·001
Physical activity index scores**							
Total physical activity	17·9	10·8	17·9	10·2	17·8	11·6	0·61
Vigorous physical activity	3·0	3·8	3·0	3·6	2·9	4·1	0·24
Moderate-to-vigorous physical activity	13·5	8·4	13·9	7·9	13·1	8·9	<0·001
Sedentary activity	11·8	6·1	11·9	6·1	11·7	6·0	0·22
Community-level characteristics							
	*n*	%	*n*	%	*n*	%	
US region							0·03
Midwest	991	19·3	577	20·3	414	18·1	
Northeast	791	15·4	451	15·9	340	14·8	
South	2135	41·6	1134	39·9	1001	43·7	
West	1221	23·8	683	24·0	538	23·5	
Income classification							0·001
High	3349	65·2	1909	67·1	1440	62·8	
Low	1789	34·8	936	32·9	853	37·2	
	Mean	sd	Mean	sd	Mean	sd	
Proportion of African Americans 5 to 14 years	19·7	23·4	17·6	21·8	22·3	25·0	<0·001
Proportion of unemployed in labour force 16 years and older	8·8	3·4	8·0	3·3	9·7	3·3	<0·001
Proportion of population with educational attainment of high school graduate or higher	80·6	10·9	83·2	10·4	77·4	10·6	<0·001

* Variables selected using the LASSO technique.

† Statistical significance was set at *P* < 0·05.

‡ Results from *χ* ^2^ tests of independence for categorical variables or t-tests for continuous variables. Multiple imputation by chained equations employed to account for missing values. Approximately a quarter (24·5 %) of cases were missing at least one variable.

§ Child race/ethnicity ‘other’ category includes Asian, Native Hawaiian/Pacific Islander, Non-Hispanic American Indian/Alaska Native, more than one race including African American and more than one race not including African American.

|| Unemployed includes those unemployed and looking for work; other includes those who are only temporarily laid off, on sick leave, or maternity leave, disabled, keeping house, retired, student and other.

¶ Graduate degree includes Master’s degree, professional degree and doctoral degree.

** Physical activity index scores represent the multiplication of the number of reported activities for each activity intensity level within the past 7 d by the frequency of participation in those activities. Greater scores indicate more physical activity and sedentary behaviours.

Close to half of children were classified as residing in a household with food insecurity, with the highest prevalence seen among Hispanic or Latino children (58·1 %) followed by non-Hispanic African American children (21·5 %) (Table 1). A high prevalence of household food insecurity was also observed in children whose parents had a maximum educational attainment of some college or Associate degree or less (87·9 %). Additionally, those living in the Southern USA (43·7 %) had higher prevalence of household food insecurity. On the community level, communities with lower income classifications, greater proportions of African American children, higher unemployment rates and fewer high school graduates also had higher prevalence of household food insecurity.

Children on average had a total physical activity index score of 17·9, a vigorous physical activity index score of 3·0, a moderate-to-vigorous physical activity index score of 13·5 and a sedentary activity index score of 11·8 (Table 1). Children in households with food insecurity had less moderate-to-vigorous physical activity than children in households without food insecurity (*P* < 0·001).

No associations were observed between household food insecurity and physical activity (Table 2). A significant interaction between food insecurity and child sex at the sedentary level was found (*P* = 0·03) (Table 3). When stratified to explore individual effects by sex, associations between food insecurity and physical activity were NS for either females or males (data not shown). There were no other associations by race/ethnicity or age (data not shown).

**Table 2:** Associations of household food insecurity and physical activity of the children in the Healthy Communities Study as measured by the 7-d Physical Activity Behaviour Recall (PABR-7) instrument (*n* 5138)

Activity type	Household food insecurity*		*P*-value†,‡
	*β*	95 % CI	
Total physical activity index score§	0·09	−0·59, 0·77	0·79
Vigorous physical activity index score||	−0·05	−0·30, 0·19	0·66
Moderate-to-vigorous physical activity index score¶	−0·12	−0·65, 0·41	0·66
Sedentary activity index score**	−0·12	−0·50, 0·27	0·55

* Binary predictor of interest was having household food insecurity (1) compared with not having (0) household food insecurity.

† Statistical significance was set at *P* < 0·05.

‡ Multilevel mixed modelling adjusted for maximum parental education, maximum parental employment status, child age, child age squared, child race/ethnicity, seasonality, US region, income classification, community percentage of African Americans, community percentage of high school graduates and community unemployment rate for those in the labour force 16 years and older. Standard errors are clustered at the community and school levels. Multiple imputation by chained equations employed to account for missing values. Approximately a quarter (24·5 %) of cases were missing at least one variable.

§ Total physical activity index score is the number of reported activities (out of 15) multiplied by the frequency of participation in those activities in the past 7 d.

|| Vigorous physical activity index score is the number of reported activities (out of 4) multiplied by the frequency of participation in those activities in the past 7 d.

¶ Moderate-to-vigorous physical activity index score is the number of reported activities (out of 11) multiplied by the frequency of participation in those activities in the past 7 d.

** Sedentary activity index score is the number of reported activities (out of 4) multiplied by the frequency of participation in those activities in the past 7 d.

**Table 3:** Moderating effect of child sex on the association between household food insecurity and physical activity of the children in the Healthy Communities Study as measured by 7-d Physical Activity Behaviour Recall (PABR-7) instrument (*n* 5138)

Activity type	Female *n* 2614 (50·9 %)				Male *n* 2524 (49·1 %)				*P*-value for interaction by sex*,†
	Without household food insecurity *n* 1390 (48·9 %)		With household food insecurity *n* 1224 (53·4 %)		Without household food insecurity *n* 1455 (51·1 %)		With household food insecurity *n* 1069 (53·4 %)		
	*β*	95 % CI	*β*	95 % CI	*β*	95 % CI	*β*	95 % CI	
Total physical activity index score‡	17·33	16·12, 18·55	17·15	15·89, 18·41	18·55	17·33, 19·77	19·04	17·78, 20·29	0·26
Vigorous physical activity index score§	2·34	1·93, 2·75	2·44	2·01, 2·86	3·52	3·11, 3·93	3·39	2·96, 3·81	0·27
Moderate-to-vigorous physical activity index score||	13·18	12·24, 14·13	12·99	12·01, 13·97	13·90	12·95, 14·85	13·91	12·93, 14·88	0·66
Sedentary activity index score¶	11·46	10·82, 12·10	11·74	11·08, 12·41	13·12	12·49, 13·76	12·69	12·03, 13·35	0·03

* Statistical significance was set at *P* < 0·05.

† Analyses used multilevel modelling to identify moderating effects of sex on the association between household food insecurity with physical and sedentary activity. Models included an interaction term for food security status (with *v*. without) by child sex (female *v*. male). Covariates included maximum parental education, maximum parental employment status, child age, child age squared, child race/ethnicity, seasonality, US region, income classification, community percentage of African Americans, community percentage of high school graduates and community unemployment rate for those in the labor force 16 years and older. Standard errors are clustered at the community and school levels. Multiple imputation by chained equations employed to account for missing values. Approximately a quarter (24·5 %) of cases were missing at least one variable.

‡ Total physical activity index score is the number of reported activities (out of 15) multiplied by the frequency of participation in those activities in the past 7 d.

§ Vigorous physical activity index score is the number of reported activities (out of 4) multiplied by the frequency of participation in those activities in the past 7 d.

|| Moderate-to-vigorous physical activity index score is the number of reported activities (out of 11) multiplied by the frequency of participation in those activities in the past 7 d.

¶ Sedentary activity index score is the number of reported activities (out of 4) multiplied by the frequency of participation in those activities in the past 7 d.

## Discussion

The results from the exploratory analysis indicate that sex may modify the relationship between household food insecurity and physical activity in children. Girls in households with food insecurity engaged in more sedentary behaviours than did girls in households without food insecurity. In contrast, boys in households with food insecurity engaged in less sedentary behaviours compared with boys in households without food insecurity. Food insecurity in the USA impacts school-age girls more than boys and is associated with poorer academic performance and higher BMI^(18,24,25)^. Additionally, participation in the Supplemental Nutrition Assistance Program is associated with better academic performance of girls more than boys, suggesting that girls may be more susceptible to some consequences of food insecurity^(26)^. Since stratification by sex did not yield significant results, additional studies are needed to better understand the influence of sex, physical activity and food insecurity.

One potential explanation for the lack of association in the main analysis lies with the food insecurity indicator used. In this study, household food insecurity was assessed by two items from the US Department of Agriculture’s eighteen-item measure that identifies households experiencing uncertainty about food access. A previous study demonstrated that the two-item tool was sensitive, specific and valid when tested in a sample of 30 098 families with young children of which 23 % were food insecure^(17)^, but these two items may not capture the experience of all individuals in the household, particularly children. Although parents attempt to protect their children from food insecurity, children are often aware of food insecurity and take responsibility for it^(27)^. Children’s experiences of food insecurity are often not known by parents^(27)^. Many children have greater awareness of household food insecurity than parents assume, and child report of their food insecurity is more strongly associated with outcomes than parent report^(28)^. Perhaps there is a relationship between food insecurity and physical activity in children, but the use of this two-item household food insecurity indicator was unable to detect this association by not assessing child experiences of food insecurity.

Previous studies that assessed the relationship between food insecurity and physical activity among children have also not used food insecurity measures that specifically assess children, such as the five-item questionnaire used in the US National Health and Examination Survey^(8,29)^ and the US Department of Agriculture’s eighteen- or six-item measures, which may have contributed to the mixed findings^(9,30)^. Using measures that specifically assess child food insecurity may be able to more accurately capture this relationship. The US Child Food Security Survey Module is one validated child-report measure for older children aged 12 to 17 years^(31)^, but it is not recommended for use in younger children. Additionally, because it was adapted from selected items in the US Household Food Security Survey Module for adults, some researchers suggest that it reflects adult experiences of food insecurity that some children may not share^(7,32,33)^. The Child Food Security Assessment is another self-reported measure that is validated for use in children as young as 6 years of age. Unlike the US Household and Child Food Security Survey Modules, the Child Food Security Assessment was developed based on interviews with children and assesses multiple subdomains of child food insecurity including cognitive awareness, emotional awareness, physical awareness, participation in parent food strategies, initiation of child food management strategies and resource generation^(32)^.

Further, given that the physical activity tool, PABR-7, was based on self-report, it may be subject to recall error. The inclusion of objective measures of physical activity, such as data collected from wearable activity tracking devices, was available but limited to 10 % of the full study sample due to the original study’s budgetary constraints^(19)^. The tool has not yet been validated against accelerometry. Additionally, although the PABR-7 focused on activities targeted by community programmes and policies, which could exclude other important forms of physical activity, it was structured similar to other physical activity self-report instruments that have been widely used in previous studies with children^(19)^. The tool reflects the composite of the child’s participation in multiple types of physical activity that are often offered in communities that are implementing obesity prevention programmes for children and youth. Therefore, the instrument and composite index should be reflective of the child’s engagement in activities that are often conceptualised as supporting prevention of obesity.

The Healthy Communities Study used a large, diverse sample of children and households from across the United States^(1)^ with a high prevalence of household food insecurity (44·6 %) and childhood obesity (24·8 %)^(34)^ compared with national estimates of food insecurity in households with children (19·5 %^(35)^ and 16·6 %^(36)^) and childhood obesity (17·2 % and 18·5 %)^(37)^ in 2013 and 2015, respectively. The study oversampled communities with certain criteria, including high proportions of African American, Hispanic and low-income households, to lend statistical power and relevance to populations experiencing health disparities^(38)^. While the study was conducted between 2013 and 2015 and has not been repeated since, the findings are relevant to today’s public health context as trends in food insecurity and childhood obesity in the United States remain high, especially in low-income and racially diverse populations. The coronavirus pandemic has increased the prevalence of child food insecurity to a projected 17·9 % in 2021^(39)^, and recent data suggest that food insecurity in households with children is close to a combined 40·0 % among African American and Hispanic households and 37·1 % among households with an income-to-poverty ratio under 1·0^(40)^. Additionally, childhood obesity has risen in 2017–2018 to 19·3 % and is estimated to be a combined 49·8 % among African American and Hispanic children^(37)^ and close to 38·8 % in households with incomes less than or equal to 350 % of the federal poverty level^(41)^. Given its observational design, the study is unable to determine causal relationships.

## Conclusion

This study suggests that sex may modify the relationship between household food insecurity and physical activity in children, though additional research is needed to clarify this relationship. Results from this study indicate that use of a more detailed food insecurity measure that better represents child experience may be warranted to assess how severity of child food insecurity is contemporaneously related to physical activity. Future studies may also explore more sub-group analyses by sex or design qualitative studies to better understand this relationship from the perspective of children.

## Supplementary material

For supplementary material accompanying this paper visit https://doi.org/10.1017/S1368980021002536. click here to view supplementary material
